# A comparison of the accuracy of digital breast tomosynthesis with supplementary views in the diagnostic workup of mammographic lesions

**DOI:** 10.1186/bcr2952

**Published:** 2011-11-04

**Authors:** JC Morel, A Iqbal, C Peacock, DR Evans, RK Wasan, R Rahim, J Goligher, MJ Michell

**Affiliations:** 1 King's College Hospital, London, UK

## Introduction

Supplementary views (SV) are routinely used in the diagnostic workup of mammographic lesions. Previous studies have demonstrated that digital breast tomosynthesis (DBT) increases the accuracy of classification of mammographic lesions [1]. In this study the diagnostic accuracy of SV is compared with DBT.

## Methods

The study included cases from screening assessment and symptomatic clinics requiring further mammographic workup. The cases were read by seven specialist breast radiologists. In the first session, readers read bilateral two-view full-field digital mammography (FFDM) and SV of the lesion. In the second session, at least 1 week later, readers read bilateral two-view FFDM plus one-view DBT in the same projection as the SV. Mammographic scores (1 to 5), lesion type and final outcome were recorded.

## Results

A total of 356 cases were assessed using receiver operative characteristic analysis to evaluate the difference between the two modes. For FFDM plus SV, the area under the curve (AUC) was 0.8807 (95% CI = 0.84374 to 0.91766) and for FFDM plus DBT the AUC was 0.9186 (95% CI = 0.88821 to 0.94895); difference in AUCs was 0.0379 with *P *value 0.055.

## Conclusion

These results demonstrate that the accuracy of DBT has borderline superiority to SV in the diagnostic workup of mammographic lesions.

## References

[B1] Michell MJ Iqbal A Wasan RK Douiri A Evans DR Peacock C Morel JC Lawinski CP Phase I trial to determine the performance of digital breast tomosynthesis versus two dimension digital and film-screen mammography [abstract SSQ01-02] 96th Scientific Assembly and Annual Meeting; 28 November-3 December 2010; Chicago, IL, USA. RSNA http://rsna2010.rsna.org

